# The Impact of DMARD and Anti-TNF Therapy on Functional Characterization of Short-Term T-Cell Activation in Patients with Rheumatoid Arthritis – A Follow-Up Study

**DOI:** 10.1371/journal.pone.0104298

**Published:** 2014-08-06

**Authors:** Balázs Szalay, Áron Cseh, Gergő Mészáros, László Kovács, Attila Balog, Barna Vásárhelyi

**Affiliations:** 1 Department of Laboratory Medicine, Faculty of Medicine, Semmelweis University, Budapest, Hungary; 2 First Department of Pediatrics, Faculty of Medicine, Semmelweis University, Budapest, Hungary; 3 Department of Rheumatology, Faculty of Medicine, Albert Szent-Györgyi Health Center, University of Szeged, Szeged, Hungary; 4 MTA-SE, Pediatrics and Nephrology Research Group, Budapest, Hungary; University of Ulm, Germany

## Abstract

Rheumatoid arthritis (RA) is a chronic autoimmune disease characterized by a systemic dysfunction of T-cells. In this study we tested the impact of DMARD and anti-TNF agents on short-term activation characteristics of T-cells. We enrolled 12 patients with newly diagnosed RA (naïve RA) who were treated with methothrexate (MTX) and glucocorticsteroid (GCS) and 22 patients with established RA non responding to conventional DMARD therapy who were treated with different anti-TNF agents. Nine healthy volunteers served as controls. Blood samples were taken at baseline, then at 4^th^ and 8^th^ week of therapy. The characteristics of several intracellular activation processes during short-term activation of T-cells including cytoplasmic Ca^2+^ level, mitochondrial Ca^2+^ level, reactive oxygen species (ROS) and nitric oxide (NO) generation were determined by a novel flow-cytometry technique. At baseline, the tested processes were comparable to controls in naïve RA. During GCS therapy, cytoplasmic Ca^2+^ level and ROS generation decreased. After the addition of MTX to GCS cytoplasmic Ca^2+^ level became comparable to controls, while ROS generation decreased further. In DMARD non responders, cytoplasmic Ca^2+^ level was higher than controls at baseline. The cytoplasmic Ca^2+^ level became comparable to controls and ROS generation decreased during each of the three anti-TNF-α agent therapies. Mitochondrial Ca^2+^ level and NO generation were unaltered in all of the patient groups. These results indicate that intracellular machinery is affected in T-cells of RA patients. This may alter the behavior of T-cells during activation. Different therapeutic approaches may modulate the abnormal T-cell functions.

## Introduction

Rheumatoid arthritis (RA) is the most common chronic autoimmune joint disease [1]. It has long been recognized that the immune phenotype (i.e. the prevalences of T-cell subsets) in RA is altered both in the synovium and in the peripheral blood [2], [3]. Besides the alterations observed in the immune phenotype, there is increasing evidence in support of the notion that abnormal lymphocyte functions also contribute to the pathogenesis of RA. Specifically, an increased level of nitric oxide (NO) production [4], altered Ca^2+^ signaling [5] and the enhanced production of reactive oxygen species (ROS) [6] during activation are hallmarks of a T lymphocyte dysfunction in RA.

Several types of traditional disease-modifying anti-rheumatic drugs (DMARDs) and immunesuppressive agents have been demonstrated to influence the T-cell subset distribution and function, and it is increasingly acknowledged that this action may contribute to the therapeutic effects of such drugs [7]. Recently, we investigated in a follow-up study the changes in T-cell subsets in RA patients in response to conventional DMARD and biological therapies [8], but intracellular activation characteristics in response to these therapies are lacking at present. Moreover, no data are available as to whether the effects on T-cell function differ between individual types of anti-TNF-α agents. We have, therefore, performed a comprehensive follow-up investigation of a variety of T-cell functional parameters including cytoplasmic Ca^2+^ level, ROS generation, NO production and mitochondrial Ca^2+^ level in activated T-cells before and during the administration of synthetic DMARDs or three different anti-TNF-α agents [adalimumab (ADA), etanercept (ETA) and infliximab (IFX)].

## Materials and Methods

### Patients

Twelve patients with naiv RA and twenty two patients with established RA were enrolled in the study. The detailed clinical data and patient characteristics are presented in Table 1.

**Table 1:** Clinical data and patient characteristics.

	Age (years)	Gender(male/female)	Disease duration(years)	Rheumatoidfactor (IU/ml)	Anti-MCV(IU/ml)	Timepoint	DAS-28index	CRP(mg/l)	ESR(mm/h)
**Naïve RA (n = 12)**	56 [50–61]	5/7	0.3 [0.2–0.3]	104.7 [41.8–138.0]	65.1 [10.3–393.6]	**Baseline (without therapy)**	6.8 [6.1–7.1]	63.8 [26.4–96.6]	64 [35–91]
						**Week 4, after medium-dose** **GCS therapy**	4.0 [2.9–4.2]	2.8 [1.4–6.8]	14 [9]–[21]
						**Week 8, after low-dose** **GCS and MTX therapy**	2.4 [2.1–3.2]	3.4 [1.6–5.3]	19 [14]–[27]
**Active RA with IFX** **therapy (n = 7)**	54 [47–57]	4/3	10.0 [4.0–14.0]	110.0 [28.6–392.2]	17.4 [4.8–395.3]	**Baseline (on** **LF and MTX therapy)**	6.2 [5.7–6.8]	13.7 [11.8–19.7]	35 [30–48]
						**Week 4 of IFX** **and MTX therapy**	5.1 [4.5–5.6]	3.1 [2.1–6.9]	17 [13–29]
						**Week 8 of IFX** **and MTX therapy**	4.1 [3.2–4.5]	2.0 [1.6–2.4]	17 [11]–[20]
**Active RA with ETA** **therapy (n = 7)**	53 [51–61]	0/7	6.5 [3.8–11.3]	85.0 [22.9–236.7]	600.0 [39.7–1000]	**Baseline (on LF** **and MTX therapy)**	6.3 [6.2–6.8]	20.1 [12.7–54.4]	54 [44–62]
						**Week 4 of ETA** **and MTX therapy**	3.3 [2.6–4.4]	3.8 [2.0–5.7]	25 [14–29]
						**Week 8 of ETA** **and MTX therapy**	2.6 [2.4–2.8]	2.0 [2.0–5.9]	13 [11–36]
**Active RA with ADA** **therapy (n = 8)**	55 [52–59]	1/7	9.0 [7.3–10.0]	187.0 [102.3–377.3]	354.6 [71.0–857.9]	**Baseline (on LF** **and MTX therapy)**	6.1 [6.0–6.5]	29.7 [18.6–43.7]	46 [39–56]
						**Week 4 of ADA** **and MTX therapy**	3.4 [3.0–4.4]	9.4 [3.1–20.4]	28 [19–37]
						**Week 8 of ADA** **and MTX therapy**	3.4 [2.9–3.7]	5.2 [2.0–8.6]	26 [17–39]

GCS = glucocorticosteroid; MTX = methotrexate; LF = leflunomide; IFX = infliximab; ETA = etanercept; ADA = adalimumab; RA = rheumatoid arthritis; Anti-MCV = anti-mutated citrullinated vimentin; DAS-28 = disease activity score in 28 joints; CRP = c-reactive protein; ESR = erythrocyte sedimentation rate.

Data are expressed as median [interquartile range].

The naïve RA group (n = 12) had not received any treatment prior to our study. After the established RA diagnosis, DMARD therapy was initiated according to a fixed protocol, which was in agreement with current EULAR and Hungarian National treatment guidelines [9]. This included the administration of medium-dose oral glucocorticosteroid (GCS, methylprednisolone, 16 mg/day) alone for 4 weeks; then GCS was tapered to 8 mg/day and methotrexate (MTX) was started with a dose of 10 mg/week. Blood samples were taken before the initiation of DMARD therapy (baseline), then after 4 and 8 weeks of treatment (i.e. after 4 weeks on medium-dose GCS and after a further 4 weeks of combination therapy with tapered-dose GCS+MTX).

In the DMARD nonresponding group with established RA (n = 22) unresponding to standard DMARD combination therapy [i.e. MTX at 15 mg/week and leflunomide (LF) at 20 mg/day], anti-TNF-α therapy was initiated following the standard-of-care decision of the treating physician: ADA at 40 mg/2 weeks sc (n = 8); ETA at 50 mg/week sc (n = 7); or IFX on week 0, 2 and 6 at 3 mg/kg iv (n = 7). At this time, LF was discontinued, while MTX was given simultaneously. Blood samples were taken before the initiation of each anti-TNF-α agent (baseline), and then on Week 4 and 8 of therapy just before the administration of the actual dose of the drug. There were no differences in the baseline characteristics (age, disease duration, disease activity, rheumatoid factor etc.) of the patients in the different anti-TNF-α groups.

The nine age- and gender-matched healthy volunteers who served as controls had a negative history of rheumatic symptoms and a negative status upon detailed physical and laboratory examination. Written informed consent was obtained in advance from all participants. The project was approved by the Ethical Committee of the University of Szeged (ETT-TUKEB 905/PI/09). This study was conducted in full accordance with the Declaration of Helsinki (1964).

### Cell preparation

Similarly to our previous study [10], 18 ml of lithium-heparin-anticoagulated blood was taken from all participants for the investigation of T-cells. Peripheral blood mononuclear cells (PBMCs) were separated by gradient centrifugation with Ficoll-Paque (GE Healthcare Life Sciences, Pittsburgh, PA, USA), washed twice with phosphate-buffered saline pH 7.4 (PBS; Central Pharmacy of Semmelweis University, Budapest, Hungary). The PBMCs were resuspended in modified RPMI medium (Sigma–Aldrich, St. Louis, MO, USA), the Ca^2+^ concentration of which was set to 2 mM. Samples were incubated with Fluo 3 AM, Rhod2 AM, dihydroethidium and DAF-FM diacetate (Molecular Probes, Carlsbad, CA, USA), which are sensitive to the cytoplasmic Ca^2+^ level, the mitochondrial Ca^2+^ level, the concentration of ROS and the level of nitric oxide, respectively. The staining conditions were identical to those reported previously [11]. Fluorescence signals were monitored for up to 10 min after the addition of phytohaemagglutinin (PHA) (Sigma–Aldrich, St. Louis, MO, USA), a non-specific activator of T-cells, in a final concentration of 20 µg/ml.

Cells were also stained with dyed antibodies against CD4 markers (Becton Dickinson, San Diego, CA, USA) allowing us to gate the cell populations to be monitored during activation.

### Equipment and statistical analysis

**Figure 1:**
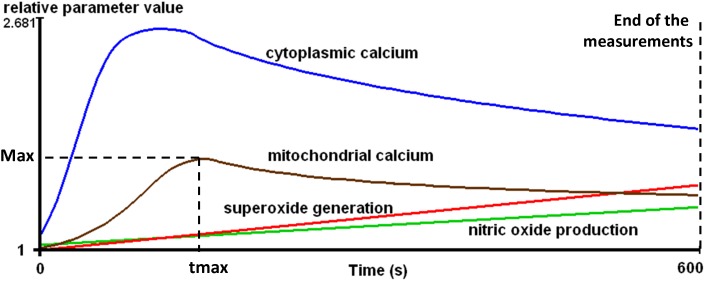
Schematic demonstration of kinetic parameters of the investigated intracellular processes during the activation of T-lymphocytes. Max = Maximum value, tmax = Time to reach maximum.

All measurements were performed on a BD FACSAria flow cytometer (Becton Dickinson, San Jose, CA, USA). The kinetic parameters of intracellular processes were determined by using R (R Foundation for Statistical Computing, Vienna, Austria) as follows. The measurement time-frame was divided into 100 time intervals of equal length and the median values were calculated in each interval. The smoothing method of Lowess was applied to the median values and each value was referred to that measured at the beginning of the experiment, furnishing the relative parameter value, rpv. The following parameters were calculated from the rpv data: area under the curve (AUC), the maximum value (Max) and the time required to reach Max (tmax) (Figure 1). One unit (U) of the AUC is defined as a rpv of 1 in 1second. Detailed explanation of these parameters is published in a previous study [12].

Further statistical analysis based on the values of these parameters was performed with R and the Statistica 7 software package (Statsoft, Tulsa, OK, USA). The Mann-Whitney test was applied for the comparison of the data on the controls and the patients, while the paired data in each related patient group were compared by the Friedman test. When the Friedman test demonstrated significant differences, the post hoc Dunn test was used to identify which pairs were significantly different. Levels of p<0.05 were taken as statistically significant. The clinical data in Table 1 and the results in Table 2 are given as median [interquartile range].

**Table 2:** Functional characteristics of intracellular processes in CD4+ cells following PHA activation in RA patients before and during therapy.

		Cytoplasmic Ca^2+^			Reactive oxygen species generation			Nitric oxide production	Mitochondrial Ca^2+^
		AUC (U)	Max (rpv)	t_max_ (s)	AUC (U)	Max (rpv)	t_max_ (s)	AUC (U)	AUC (U)
**Control (n = 9)**		89.06 [62.60–131.5]	1.211 [1.149–1.326]	444.8[192.3–594.4]	75.43[68.01–88.80]	1.239[1.217–1.263]	594.5[594.3–595.3]	5.501[−6.919–35.98]	64.18[42.85–97.89]
**Naïve RA (n = 12)**	**Baseline** **(without therapy)**	90.54 [73.45–206.5]	1.221 [1.166–1.581]	458.9[172.6–594.5]	74.36[50.26–90.26]	1.245[1.178–1.308]	594.5[594.0–595.2]	34.11[−0.103–76.15]	63.03[33.70–97.45]
	**Week 4, after** **medium-dose** **GCS therapy**	44.55^A.B^ [21.11–74.37]	1.127^A^ [1.068–1.201]	234.2[209.0–594.1]	48.47^A.B^[36.15–79.09]	1.172[1.145–1.237]	594.7[594.3–595.4]	−9.670[−11.43–10.73]	40.11[25.71–58.89]
	**Week 8, after** **low-dose GCS** **and MTX therapy**	60.67 [12.22–111.6]	1.232 [1.046–1.295]	306.5[289.5–594.1]	49.75^A^[45.87–54.55]	1.170^A^[1.165–1.179]	594.6[594.5–594.8]	3.180[−12.39–11.74]	55.83[19.47–72.22]
**Active RA with IFX** **therapy (n = 7)**	**Baseline (on LF** **and MTX therapy)**	145.1^A^ [126.8–207.4]	1.374^A^ [1.315–1.510]	594.1[342.8–594.6]	65.04[53.47–86.19]	1.223[1.170–1.258]	594.6[594.4–595.2]	24.62[−7.701–95.59]	51.86[44.98–81.62]
	**Week 4 of IFX** **and MTX therapy**	96.97 [4.403–162.7]	1.240 [1.032–1.352]	594.5[276.2–594.6]	39.92^A.B^[33.42–67.93]	1.146^A^[1.141–1.230]	594.4[593.7–595.2]	19.82[−14.59–30.06]	83.87[51.44–166.5]
	**Week 8 of IFX** **and MTX therapy**	115.9 [59.33–141.1]	1.328 [1.242–1.347]	593.9[288.0–594.9]	56.16^A^[45.14–71.85]	1.186^A^[1.167–1.220]	593.6[593.1–595.5]	−2.549[−16.00–25.92]	67.37[42.57–118.9]
**Active RA with ETA** **therapy (n = 7)**	**Baseline (on LF** **and MTX therapy)**	141.2^A^ [109.1–235.6]	1.319^A^ [1.271–1.535]	372.0[216.7–593.5]	94.55^A^[81.39–111.0]	1.293[1.242–1.372]	594.9[594.2–595.6]	−0.087[−12.70–22.17]	70.62[59.08–128.8]
	**Week 4 of ETA** **and MTX therapy**	125.7 [64.09–159.1]	1.304 [1.147–1.396]	383.8[246.1–594.1]	73.49^B^[59.69–81.87]	1.235^B^[1.188–1.275]	594.8[594.1–595.3]	21.72[−4.121–87.42]	50.93[34.75–82.80]
	**Week 8 of ETA** **and MTX therapy**	92.11 [49.82–222.8]	1.231 [1.182–1.491]	312.5[174.3–593.9]	72.37[49.84–105.9]	1.226[1.163–1.300]	593.9[593.7–594.8]	8.466[−8.267–62.04]	77.45[26.15–94.89]
**Active RA with ADA** **therapy (n = 8)**	**Baseline (on LF** **and MTX therapy)**	144.4^A^ [128.0–199.8]	1.399^A^ [1.320–1.484]	557.9[217.8–594.4]	81.89[69.52–106.8]	1.258[1.220–1.343]	594.5[594.0–594.9]	15.86[1.704–116.9]	77.94[66.09–90.10]
	**Week 4 of ADA** **and MTX therapy**	93.91 [77.45–157.3]	1.227 [1.188–1.347]	497.4[256.4–594.6]	63.21^B^[34.48–77.15]	1.212[1.155–1.240]	594.7[594.2–595.5]	25.87[−6.588–99.43]	66.84[41.83–92.19]
	**Week 8 of ADA** **and MTX therapy**	107.6 [50.18–158.5]	1.262 [1.146–1.364]	432.4[226.6–594.5]	73.95[61.69–78.82]	1.230[1.211–1.241]	594.2[593.6–594.9]	60.43[26.93–133.0]	71.77[62.20–95.48]

Data are expressed as median [interquartile range].

A versus control P<0.05;

B versus baseline P<0.05.

AUC = area under the curve; Max = maximum value; t_max_ = time to reach maximum value; rpv = relative parameter value.

GCS = glucocorticosteroid; MTX = methotrexate; LF = leflunomide; IFX = infliximab; ETA = etanercept; ADA = adalimumab; RA = rheumatoid arthritis.

## Results

### Naïve RA patients subjected to DMARD therapy

**Figure 2:**
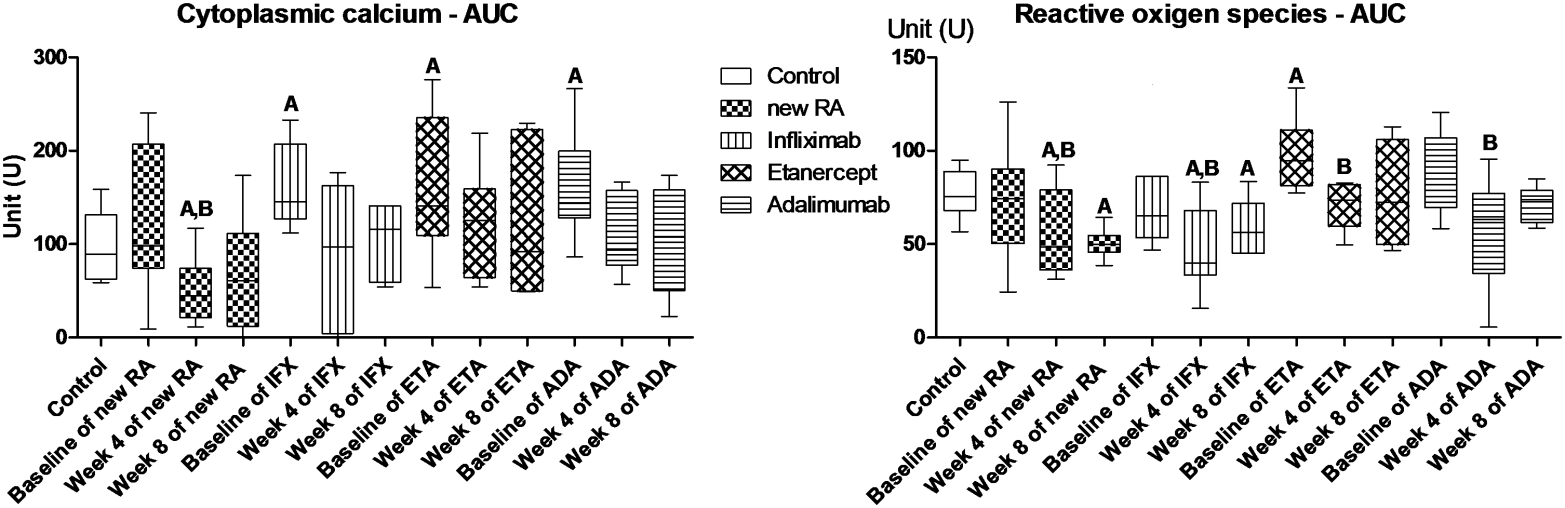
Area under the curve values of cytoplasmic calcium and reactive oxigen species in CD4+ cells. A-versus controls P<0.05; B-versus baseline P<0.05. AUC = area under the curve; RA = rheumatoid arthritis; IFX = infliximab; ETA = etanercept; ADA = adalimumab.

At baseline, the investigated intracellular parameters of the T-cells during short-term activation were similar to those of the controls (Table 2, Figure 2). At Week 4 of GCS therapy, the cytoplasmic Ca^2+^ response and the level of ROS production during T-cell activation had decreased significantly and become lower compared to the controls and the baseline values. At Week 8, cytoplasmic Ca^2+^ response had become comparable to the baseline and control levels, while the ROS production was further impaired.

### DMARD nonresponder patients subjected to anti-TNF-α agents

At baseline, the cytoplasmic Ca^2+^ level was higher in this patient group than in the controls (Table 2, Figure 2). The other parameters investigated, including the mitochondrial Ca^2+^ levels, ROS and NO production were comparable to those observed in the controls with the exception of the subgroup treated later with ETA. (These patients exhibited an increased ROS generation capacity at baseline).

On Week 4 of anti-TNF-α therapy, the cytoplasmic Ca^2+^ level became comparable to controls, while ROS generation was decreased significantly to below the baseline, irrespective of the nature of the anti-TNF-α agent administered (including ETA). At this time point, the other parameters investigated were still comparable to those at the baseline in each patient group.

At Week 8, no significant alterations were detected relative to Week 4 in any of the parameters tested.

## Discussion

This is the first study that provides a follow-up of T-cell activation characteristics in RA patients subjected to different therapeutic regimes. Our study involved two different RA groups: naïve RA and DMARD-non responding patients.

### Naïve patients

There is a paucity of studies investigating T-cell activation characteristics in RA. The few data available suggest that increased levels of nitric oxide [13], intracellular Ca^2+^ [4] and ROS [6] are present in the T-cells of active RA patients and these abnormalities may contribute to the cellular dysfunction. However, in naïve RA patients we found that these parameters were comparable to those in the controls. The discrepancy between our results and previous studies could be due to the different populations tested as we enrolled newly diagnosed naïve RA subjects, while the published data involved a less specific RA population. Indeed, as we discovered in the case of patients who had suffered from RA for several years, these intracellular mechanisms may be affected in the later stage of the disease that does not respond to DMARD therapy.

The cytoplasmic Ca^2+^ response and ROS production during the short-term activation of T lymphocytes were suppressed relative to the baseline after 4 weeks of GCS therapy. In *in vitro* and *in vivo* animal studies GCS inhibited activated T-cell functions at several points of the intracellular signaling cascade [14]. GCS treatment limits PIP2 hydrolysis and IP3 production [15], down-regulates IP3 receptors [16] and also depletes internal Ca^2+^ stores [17]. Irrespective of the mechanism, the result at a cellular level is the reduction of the Ca^2+^ responses during short-term T-cell activation. GCS therapy also decreased intracellular ROS levels in human aortic smooth muscle cells [18]. These reported data are in accordance with our results indicating that GCS therapy has a major impact on the T-cell functionality in early RA.

After 1 month of MTX treatment, the cytoplasmic Ca^2+^ response during T-cell activation became comparable to controls. An in vitro animal study revealed evidence that MTX has IP3-like properties and mobilizes Ca^2+^ from the endoplasmic reticulum in a direct way without receptor activation [19]. The increase in the cytoplasmic Ca^2+^ level may reflect this effect (while it is still uncertain whether the normal appearance of the cytoplasmic Ca^2+^ level does indicate normal Ca^2+^ signaling in this situation).

In MTX-treated subjects, ROS generation was suppressed further. This phenomenon confirms previous results observed on RA synoviocytes, where MTX inhibited the production of ROS [20]. This finding may be of major clinical relevance, as excessive ROS production plays a critical role in the pathogenesis of RA [21]. Besides the role of extracellular ROS generation, it has also been demonstrated that increased intracellular ROS production amplifies the synovial inflammatory–proliferative response [22] and is characteristic of and augments the T-cell dysfunction in RA [23].

### RA patients unresponding to DMARD therapy

In the second part of our study, we monitored T-cell functionality of RA patients on anti-TNF-α therapy. We also investigated whether different agents procure different effects on these parameters.

At baseline, (i.e. just before anti-TNF-α therapy) we found a higher cytoplasmic Ca^2+^ response to stimulation than in the controls. It was noteworthy that the increased Ca^2+^ signal became comparable to controls by Week 4 and remained unaltered at Week 8, irrespectively of the anti-TNF-α agent used. Although this occurred in almost all the patients, it is in contradiction with most of the published data that suggest hyporesponsiveness of the T-lymphocytes in RA [24], [25].

The explanation of the discrepancy between the published and our own data is probably the different characteristics of the RA patients enrolled. The enrolled patients in the published studies were predominantly treated with NSAIDs (with no reported effect on the calcium metabolism in the T-cells). In contrast, we collected samples from patients who did not respond to DMARD therapy (that included MTX and LF). No effect of LF on T-cell Ca^2+^ signaling is known, while MTX has been documented to increase Ca^2+^ response. When LF was replaced by any of the anti-TNF-α agents, the cytoplasmic Ca^2+^ signal became comparable to controls. It is unclear whether this is due to the introduction of the anti-TNF-α agent and/or to the cessation of LF. Irrespective of the mechanism, our results should be regarded as characteristic of this specific RA subgroup. It is tempting to postulate that an increased cytoplasmic Ca^2+^ signal might possibly be used as a surrogate marker for the identification of RA patients in whom combination of MTX with leflunomide will not be sufficient and leflunomide is recommended to be replaced by an anti-TNF agent. This hypothesis should be evaluated further.

While our results also indicate that NO production and mitochondrial Ca^2+^ handling are not affected during short-term T-cell stimulation by anti-TNF agents in RA, we observed changes in intracellular ROS production during anti-TNF therapy. As discussed above, an increased intracellular ROS production may contribute to the prolonged inflammation observed in RA, and a reduction of ROS may therefore be a beneficial effect. We also found that the baseline ROS production was higher in the ETA-treated group and tended to be higher in the ADA group than in the controls. Although the overproduction of TNF-α is thought to be the main contributor to the increased ROS generation in RA, the effects of anti-TNF-α treatment have not been extensively studied in this RA patient subgroup.

The effect of anti-TNF agents on T-lymphocyte ROS production is not fully elucidated. Data are limited to neutrophils. Pay et al reported that IFX inhibited the production of ROS in neutrophils *in vitro* [26], while anti-TNF-α treatment has been stated to be ineffective on ROS production of the neutrophils in RA patients [27], [28]. For T-lymphocytes, we demonstrated a significant decrease in all groups at Week 4 of anti-TNF-α therapy, irrespective of whether the baseline ROS production was higher than or comparable to the controls. This effect was most pronounced in the IFX-treated group and proved to be associated with significantly decreased ROS production. At Week 8, we found no changes in the production of ROS relative to that at Week 4; again, this type of anti-TNF action was comparable in the various treatment groups.

### The limitations of our work

Evaluating these results one should note that because of the low number of patients enrolled, these observations are suitable for just hypothesis generation and the data can not be extrapolated to large patient cohorts.

In the DMARD nonresponding patients at initiation of anti-TNF-α therapy leflunomide was discontinued, but we could not investigate whether this effects the intracellular processes in any way.

## Conclusions

This study on peripheral blood samples has provided comprehensive information regarding the functionality of T-cells in RA.

The intracellular T-cell activation processes (including cytoplasmic Ca^2+^, ROS, NO generation and mitochondrial Ca^2+^) during short-term T-cell activation were not altered in early RA. The therapeutic interventions exerted specific effects on the different parameters. GCS decreased cytoplasmic Ca^2+^ signal and the levels of ROS. Tapering the GCS dose and the introduction of MTX increased the cytoplasmic Ca^2+^ signal, but further decreased the levels of ROS. In DMARD non responding patients, the cytoplasmic Ca^2+^ level was higher than in the controls (possibly due to the treatment with MTX). After the introduction of TNF α inhibitors, the cytoplasmic Ca^2+^ signal became comparable to controls and the levels of ROS decreased. The extent of NO generation and the level of mitochondrial Ca^2+^ were not altered at any time during our study.

These results indicate that intracellular machinery is affected in T-cells of RA patients. This may alter the behavior of T-cells during activation. Different therapeutic approaches may modulate the abnormal T-cell functions.
